# Extensive duplication of the *Wolbachia* DNA in chromosome four of *Drosophila ananassae*

**DOI:** 10.1186/1471-2164-15-1097

**Published:** 2014-12-12

**Authors:** Lisa Klasson, Nikhil Kumar, Robin Bromley, Karsten Sieber, Melissa Flowers, Sandra H Ott, Luke J Tallon, Siv G E Andersson, Julie C Dunning Hotopp

**Affiliations:** Department of Cell and Molecular Biology, Molecular Evolution, Uppsala University, Uppsala, Sweden; Institute for Genome Sciences, University of Maryland School of Medicine, Baltimore, MD 21201 USA; Department of Microbiology & Immunology, University of Maryland School of Medicine, Baltimore, MD 21201 USA

**Keywords:** *Drosophila ananassae*, *Wolbachia*, Lateral gene transfer, Horizontal gene transfer, Symbiosis, Underreplication, Heterochromatin

## Abstract

**Background:**

Lateral gene transfer (LGT) from bacterial *Wolbachia* endosymbionts has been detected in ~20% of arthropod and nematode genome sequencing projects. Many of these transfers are large and contain a substantial part of the *Wolbachia* genome.

**Results:**

Here, we re-sequenced three *D. ananassae* genomes from Asia and the Pacific that contain large LGTs from *Wolbachia.* We find that multiple copies of the *Wolbachia* genome are transferred to the *Drosophila* nuclear genome in all three lines. In the *D. ananassae* line from Indonesia, the copies of *Wolbachia* DNA in the nuclear genome are nearly identical in size and sequence yielding an even coverage of mapped reads over the *Wolbachia* genome. In contrast, the *D. ananassae* lines from Hawaii and India show an uneven coverage of mapped reads over the *Wolbachia* genome suggesting that different parts of these LGTs are present in different copy numbers. In the Hawaii line, we find that this LGT is underrepresented in third instar larvae indicative of being heterochromatic. Fluorescence *in situ* hybridization of mitotic chromosomes confirms that the LGT in the Hawaii line is heterochromatic and represents ~20% of the sequence on chromosome 4 (*dot* chromosome, Muller element F).

**Conclusions:**

This collection of related lines contain large lateral gene transfers composed of multiple *Wolbachia* genomes that constitute >2% of the *D. ananassae* genome (~5 Mbp) and partially explain the abnormally large size of chromosome 4 in *D. ananassae.*

**Electronic supplementary material:**

The online version of this article (doi:10.1186/1471-2164-15-1097) contains supplementary material, which is available to authorized users.

## Background

In general, lateral gene transfer (LGT) can be defined as the transfer of DNA between organisms that does not result from sexual reproduction. It can allow organisms to acquire novel traits that are unique from those that are vertically inherited and occasionally results in higher fitness. However, since the transferred DNA has little to no effect on fitness in most cases, it is often lost from the population shortly after the transfer has occurred. Although LGT has been described in many different organisms over the last decades, relatively fewer cases have been found in animals compared to many other taxa. However, when identified in animals, the donors are often cytoplasmic inhabitants, such as mitochondria or intracellular bacteria. For example, *nu*clear *m*itochondrial *t*ransfers (numt) have been found in nearly every eukaryotic genome and typically represent 0.1% of the genome [1]. In animals, most of the LGT events identified as originating from bacteria have occurred between *Wolbachia* bacteria and their various hosts.

*Wolbachia* endosymbionts are bacteria that colonize filarial nematodes and several different arthropods, including >20% of insect species [2–4]. While obligate mutualists in nematodes, *Wolbachia* are best known for the ability to manipulate host reproduction in arthropods in various ways which include induction of parthenogenesis, feminization, male killing, and cytoplasmic incompatibility [2]. In general, *Wolbachia* bacteria are intracellular and maternally inherited through the egg cytoplasm [2, 3]. As some strains have also been shown to specifically target the germline stem cell niche [5], these endosymbionts are ideally located to bring about heritable LGT to eukaryotic genomes [6, 7], since they colonize the cells that will give rise to the germ line and therefore offspring.

*Wolbachia*-host LGT events are termed nuwts [6] for *nu*clear W*olbachia t*ransfers (pronounced: noot) following the established nomenclature for numts and nupts, *nu*clear *p*lastid *t*ransfers. The first nuwt described was found in the bean beetle *Callosobruchus chinensis*[8] that contains ~30% of a *Wolbachia* genome on the X-chromosome, and where about half of the genes examined showed low level transcription [9]. Nuwts have now been identified in the genomes of many diverse invertebrate taxa [6, 8–13] and several transfers of large segments of the *Wolbachia* genome have been described, e.g. in *Monochanus alternatus* (longicorn beetle) [14] and *Glossinia morsitans morsitans* (tsetse fly) [15]. Furthermore, ~20% of the nematode and arthropod species where whole genome sequence data is available have evidence supporting the presence of nuwts [10], and 80% of the genomes of all known *Wolbachia* hosts have some evidence indicating the presence of nuwts [10]. Interestingly, every filarial nematode genome examined thus far has been shown to contain nuwts [10, 11, 16, 17], including nematodes that currently lack a *Wolbachia* endosymbiont [16–18].

Although very little is known in general about the function of nuwts, several nuwts or nuwt-related sequences have been found that give rise to genes that are expressed and possibly functional in their new environment. For example, salivary gland genes have been transferred between mosquitoes and *Wolbachia*, possibly in both directions [19, 20] and experimental data for one of them showed specific localization of the expressed protein to the basal lamina of the salivary gland and indicated its importance for malaria sporozite invasion [21]. Furthermore, expression of twenty genes of bacterial origin from the nuclear genome in mealybugs has been demonstrated [22]. Although this does not directly infer functionality, the majority of these twenty genes has been lost in the obligate endosymbionts of the mealybug, and some of these nuclear encoded genes complement the genes retained in the endosymbionts leading to complete pathways. For example, riboflavin biosynthesis requires three insect genes that presumably arose via LGT from facultative endosymbionts belonging to the α-Proteobacteria and Bacteroidetes phyla, which include the genera *Wolbachia, Cardinium,* and *Arsenophonus*[22]. A similar result of identifying LGT from extant facultative endosymbionts was observed through an exhaustive analysis of LGT in the pea aphid, *Acyrthosiphon pisum.* That analysis revealed twelve genes or gene fragments of bacterial origin [12], five of which are most closely related to bacterial genes from the order Rickettsiales [12], of which *Wolbachia* is a member. One of these genes, *ldcA*, is transcribed in the aphid bacteriocyte and is involved in bacterial cell wall metabolism [12]. Interestingly, *Buchnera aphidicola,* an obligate mutualistic γ-proteobacterium associated with aphids, has all the components to synthesize the bacterial cell wall, except *ldcA,* and it has been hypothesized that expression of this nuclear-encoded gene in the bacteriocyte may allow aphids to control growth of its endosymbiont by manipulating biosynthesis of the endosymbiont’s cell wall [23]. Lastly, many filarial nematodes possess a ferrochelatase gene that was acquired by LGT from an α-Proteobacteria in the Rhizobiales [24]. This ferrochelatase, which encodes the terminal step of heme biosynthesis, is functionally active in an enzyme assay and complements an *E. coli hemH* mutant. In *Brugia malayi*, the ferrochelatase is involved with filarial motility and viability, as assessed with a ferrochelatase inhibitor, and is essential, as assessed with RNAi silencing with abnormalities arising in nuclei of germ cells and embryos [24].

The nuwt in *Drosophila ananassae,* examined here, was initially estimated to consist of an entire ~1.4 Mbp *Wolbachia* genome integrated on the left arm of chromosome 2 in a *D. ananassae* line from Hawaii [10]. Only low level transcription of 28 of the 1,206 nuwt genes tested was observed in the *D. ananassae* Hawaii line at the time [10], and was later confirmed by the low abundance of nuwt sequences in the transcriptome of *Wolbachia-*depleted adult flies [25]. Although, the biological relevance of this low level transcription is not known, these results did not give strong indication for functionality of the nuwt. However, in fourteen lines from the *Drosophila* Stock Center, PCR screening performed in the same study revealed the presence of similar nuwts in three more lines of *D. ananassae* from Asia and the Pacific, warranting further investigation into the evolution of this large nuwt in *D. ananassae.*

Here, we describe the results of re-sequencing three of the four *D. ananassae* lines originally discovered to contain large nuwts and the results of further *in situ* hybridization to examine its chromosome position and structure. The analysis confirmed integration in all three lines and revealed that the integrated genomes are affected by extensive duplication and underreplication. We demonstrate that in the Hawaii line the nuwt is most likely located on the heterochromatic fourth chromosome, which is abnormally large in *D. ananassae* compared to its close relatives*.* Taken together, the results suggest that the nuwt in *D. ananassae* may not currently provide any function, but rather that it is tolerated in its new genome and contributes to the large size of a chromosome that is already riddled with non-coding DNA and repeats.

## Results

### *D. ananassae*lines used for genome sequencing

Three of the four *D. ananassae* lines (Hawaii, India, and Indonesia) that were previously found to have a nuwt [10] were re-sequenced. All three lines are infected with *Wolbachia* bacteria (Wb+) and contain the nuwt (nuwt+) [10]. Because sequence data from contaminating *Wolbachia* bacteria would complicate the analysis of the nuwt, the flies were treated with tetracycline (tet) for three generations prior to DNA extraction in order to create Wb- nuwt+ lines. To further ensure the absence of *Wolbachia* DNA originating from the bacterial genome rather than the nuwt in the sequencing data, the ovaries of tet-treated flies were confirmed to be Wb- by fluorescence *in situ* hybridization (FISH) microscopy with *Wolbachia*-infected flies serving as a positive control*.* DNA for sequencing was then isolated from the remaining carcass. Separately, DNA was isolated from 3^rd^ instar larvae from a cross between a tet-treated India male and a Florida female that is naturally Wb- and nuwt-. Female F1 offspring were subsequently backcrossed with males of the parental tet-treated India line, which resulted in a line without the *Wolbachia* endosymbiont, with Florida mitochondria, and a mix of Florida and India nuclear genomes slightly enriched for India.

Despite numerous attempts to cure the Malaysia line, which is the fourth line where a nuwt was previously detected [10], we were unable to generate the necessary numbers of *Wolbachia*-free insects from this line for sequencing. On three separate instances, the line was acquired from the stock center, successfully cured using tetracycline for two generations, and the absence of *Wolbachia* endosymbionts verified. However, following curing, the lines would not proliferate to the needed levels. It is not clear if this is due to the absence of *Wolbachia,* the absence of other members of the microbiome, or other factors. However, as a result, this line was not sequenced.

### Genome sequencing and mapping

The DNA extracted from each line was used to construct an ~3-kbp Illumina mate pair library and an ~300-bp Illumina paired end library. The resulting reads were mapped against the ~231 Mbp *D. ananassae* caf1 assembly [26] and the ~1.4 Mbp *w*Ri *Wolbachia* genome [27] using BWA [28]. The *w*Ri genome was chosen as the reference for further analyses because (a) previous analyses have shown that *w*Ri is more than 99% identical to the *Wolbachia* sequences obtained from the *D. ananassae* genome sequencing project [27, 29, 30], (b) the mapped reads cover the whole *w*Ri genome, including genes that are not shared between *w*Ri and *w*Mel [27], and (c) the *Wolbachia* sequences in the selected *D. ananassae* lines are estimated to differ by less than one SNP in 20,000 bp [31]. Thus, the *w*Ri genome should be useful as a reference for the *Wolbachia* reads in all of the sequenced *D. ananassae* genomes.

We considered the risk that mitochondrial sequences might erroneously map to the *w*Ri genome. Across all three datasets, only nine reads of the more than 500,000 reads were identified that mapped to both locations (1 in Hawaii, 0 in India, and 8 in Indonesia). Given these very low numbers that would not influence the values presented nor the interpretation, we did not consider cross mapping an issue to address further. The only regions where we saw erroneous mapping of reads were in the rRNA genes (see below), which likely arose because fed adults with an active gut microbiome were used for preparing genomic DNA.

### Analysis of sequence coverage for the Hawaii line

**Figure 1:**
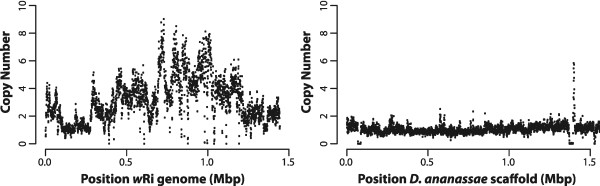
**Coverage of Hawaii sequencing data.** The copy number for the 3 kbp mate pair library from the Hawaii genomic DNA was calculated over a 1 kbp window every 500 bp and plotted against the reference *w*Ri genome (left) and the first 1.5 Mbp of the largest scaffold (gi|109914400|gb|CH902617.1|) of the *Drosophila ananassae* caf1 assembly. Approximately 5× coverage was expected to represent single copy *Drosophila* genes and single copy regions are apparent. However, most of the nuwt is present with at least two copies.

Approximately 5× coverage of the *Drosophila* nuclear genome was expected for single copy genes based on the sequencing output of the mate pair library of the Hawaii line. Although mapping of the reads from this library to the *D. ananassae* genome shows the expected 5× coverage, or 1 copy (Figure 1), when mapping the same dataset against the *w*Ri genome, the coverage varies, suggesting 1–8 copies, with the majority of the genome having ≥10× coverage, or 2 copies (Figure 1). This suggests extensive duplications of the nuwt in this line with up to 12 copies per haploid genome in regions where the sequencing coverage is 60×.

**Figure 2:**
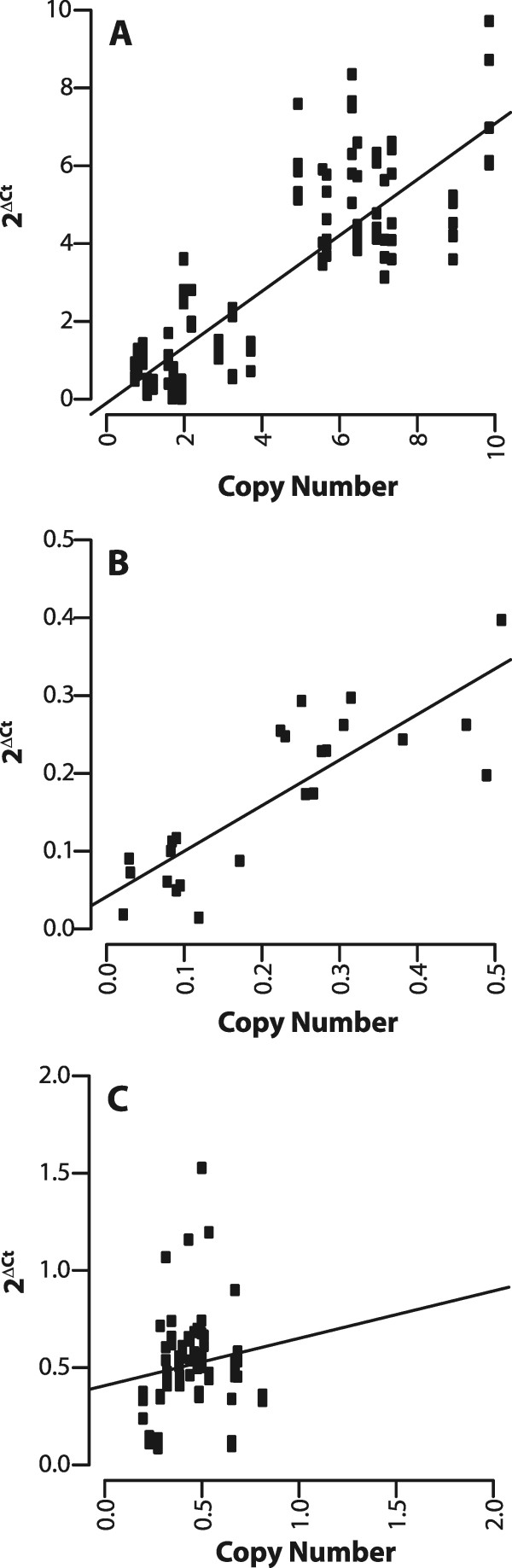
**Confirmation of coverage metrics using a qPCR based assay.** The copy number was confirmed and found to be accurate up to 8 copies per haploid genome using a qPCR assay measuring the ratio of the LGT abundance to that of six single copy *Drosophila* genes as shown for the 3 kbp mate pair library for the cured Hawaii **(Panel A)**, India **(Panel B)**, and Indonesia **(Panel C)**. A significant positive correlation (p < 0.001, linear regression) is found for the cured Hawaii and the India line The results in Panels **A** and **C** represent the accumulation of multiple qPCR experiments while those in Panel **B** result from a single experiment.

Previously, similar copy number differences were observed using Illumina sequence reads and were successfully validated for the nuwts in *B. malayi*[32]*.* Therefore, a qPCR assay was developed using 24 different primer pairs that amplify 4–5 regions predicted to be at approximately 1, 2, 4, 8, and 12 copies in the nuwt of the Hawaii line. In this assay, relative abundance is estimated by measuring the difference between the cycle threshold (Ct) values, i.e. the number of PCR cycles required for the fluorescent signal to exceed the background level, for the amplicons in the nuwt relative to the average Ct value of 6 single copy *D. ananassae* genes to yield the ΔCt value (Ct(nuwt amplicon)-Ct(average *D. ananassae* genes). The ratio of the abundance between the nuwt amplicon region and the average single copy gene is then 2^ΔCt^ and should equal the number of copies of the nuwt amplicon in the genome as determined by genome sequencing. As expected, 2^ΔCt^ has a positive linear correlation with the copy number over the length of the amplicon (Figure 2A) for the Hawaii line, where up to 8 gene copies per haploid genome could be validated. This suggests there may be as many as 16 copies per nucleus of large portions of this nuwt.

### Analysis of sequence coverage for the India line

**Figure 3:**
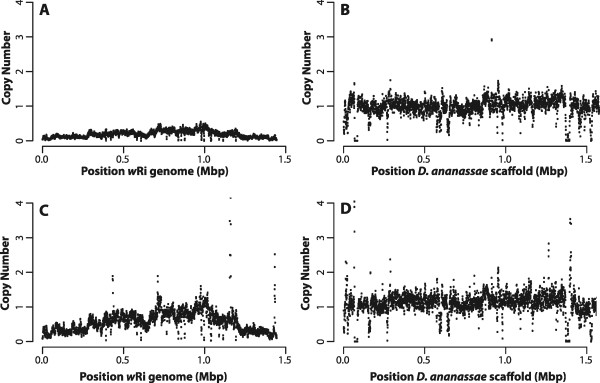
**Coverage of India sequencing data.** The copy number for the 3 kbp mate pair library from the India **(Panels A and** **B)**, and India x Florida **(Panels C and** **D)** genomic DNA was calculated over a 1 kbp window every 500 bp and plotted against the reference *w*Ri genome (left) and the first 1.5 Mbp of the largest scaffold (gi|109914400|gb|CH902617.1|) of the *Drosophila ananassae* caf1 assembly. The India genome shows the same relative pattern of duplication but is less abundant than single copy nuclear genes, as is the genome from offspring of a backcross of India with Florida. The spikes in the coverage for the India × Florida line reflect the mapping of transposase sequences.

**Figure 4:**
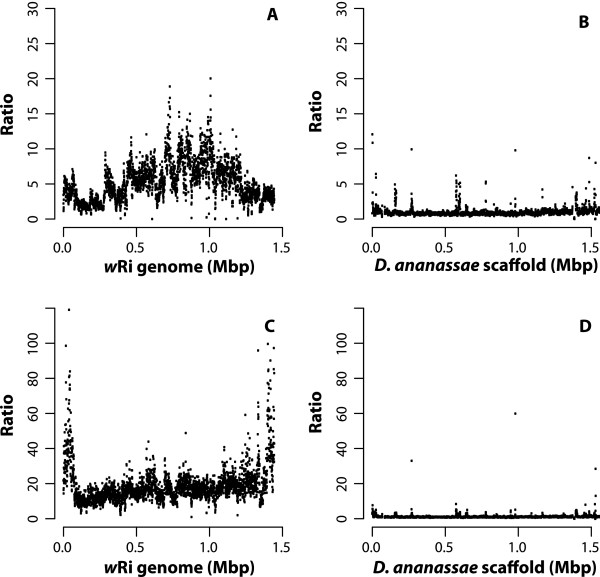
**Comparison of the coverage patterns.** The coverage of the Indonesia genome is shown relative to that of the Hawaii line by dividing the values for Hawaii line by those of the Indonesia line across the *w*Ri genome **(Panel A)** and the *Drosophila ananassae* genome **(Panel B)**. The coverage of the India line relative to the Hawaii line is illustrated in the same manner **(Panels C and** **D)**. While the India line has the same pattern of duplication as Hawaii, that pattern is different in the Indonesia line.

When performing the same analysis on the India line, a consistently lower coverage over the nuwt is observed when compared to single copy *Drosophila* genes (Figure 3), suggesting <1 copy per haploid genome. Even so, India has a highly similar pattern of coverage variation to the one observed in the Hawaii line, indicating the presence of similar duplications (Figure 4). This result was consistent in three independent sequencing runs of (a) third instar larvae from the cross between India and Florida (I × F) maintained by LK and sequenced at NCGR, Santa Fe, NM (Table 1); (b) third instar larvae from I × F maintained by LK and sequenced at IGS from DNA isolated on a separate occasion (Table 1, Figure 3); and (c) adult flies from a tet-treated India line obtained from the stock center at a separate time and maintained in the JCDH lab and sequenced at IGS (Table 1, Figure 3). This sequence coverage was validated with the qPCR assay described above, and a correlation similar to that for the Hawaii line was observed (Figure 2B).

**Table 1:** **Summary of the sequencing and mapping statistics**

Line	Strategy	Combined read length	Reads sequenced	*Drosophila* reads mapped	*w* Ri reads mapped	Ratio of *w* Ri reads to *Drosophila* reads
Hawaii	MP	80	33,602,456	26,875,741 (80.0%)	605,174 (1.8%)	2.3%
Hawaii	PE	129	55,650,278	47,522,893 (85.4%)	471,055 (0.85%)	0.99%
IndiaxFlorida	MP	108	108,816,304	16,398,509 (15.1%)*	258,215 (0.09%)	0.57%
IndiaxFlorida	MP	72	62,988,324	40,744,911 (64.6%)	183,582 (0.29%	0.45%
IndiaxFlorida	PE	202	165,515,736	150,307,807 (90.8%)	702,594 (0.42%)	0.46%
India	MP	80	64,363,460	40,552,454 (63.0%)	67,983 (0.11%)	0.17%
India	PE	202	90,067,451	83,518,157 (92.7%)	88,597 (0.098%)	0.11%
Indonesia	MP	70	72,675,508	40,576,655 (55.8%)	185,889 (0.26%)	0.46%
Indonesia	PE	150	64,503,996	51,912,428 (80.5%)	258,215 (0.40%)	0.40%

The sequencing statistics for line and sequence sequence strategy are provided, including the combined read length, number of reads sequenced, number of non-duplicate reads mapping to the *D. ananassae* caf1 assembly, number of non-duplicate reads mapping to the *w*Ri genome, and the ratio of the reads mapping to the *w*Ri and *D. ananassae* genomes.

*This library had a significant number of duplicate mate pairs such that >84% of reads mapped, but >80% of mapped reads were duplicates.

### Analysis of sequence coverage for the Indonesia line

**Figure 5:**
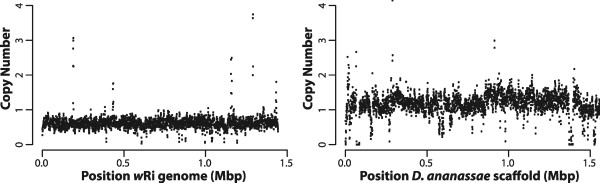
**Coverage of Indonesia sequencing data.** The copy number for the 3 kbp mate pair library from the Indonesia genomic DNA was calculated over a 1 kbp window every 500 bp and plotted against the reference *w*Ri genome (left) and the first 1.5 Mbp of the largest scaffold (gi|109914400|gb|CH902617.1|) of the *Drosophila ananassae* caf1 assembly. The Indonesia line has a nuwt with even coverage similar to the rest of the *Drosophila* genome. The spikes in the coverage for the Indonesia line reflect the mapping of conserved DNA sequences (e.g. those from rRNA or tRNA) from bacterial contaminants like those in the guts of adult flies.

In contrast to Hawaii and India, the Indonesia line has very even sequence coverage, indicating that there is no variation in copy number between different regions of the nuwt in this line (Figure 5). As expected, Indonesia does not show a positive linear correlation using the qPCR assay since all of the genes have the same copy number. Therefore, the plot only shows the random variation present in the data (Figure 2C) supporting the assessment of coverage using the sequence read mapping. In all three cases examined, paired end reads yield approximately the same results as mate pair reads (data not show).

### Copy number in the presence of the *Wolbachia*endosymbiont

**Figure 6:**
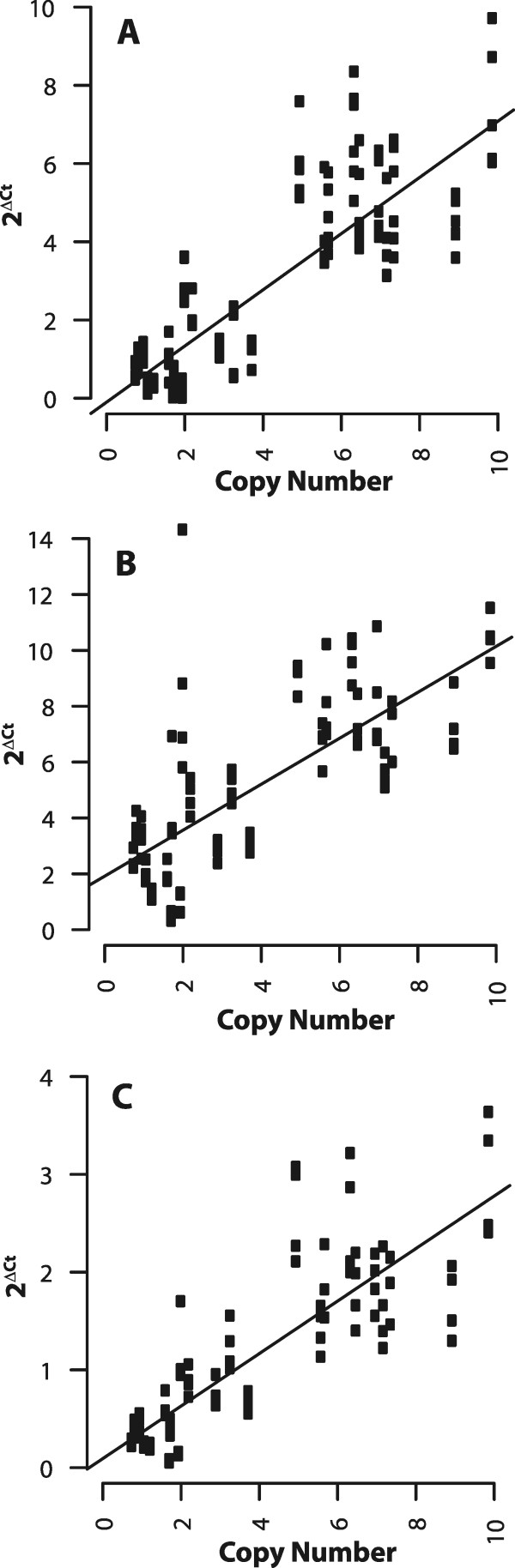
**Comparison of copy number of the nuwts in homozygous and heterozygous fly lines and cured v. uncured flies.** Cured **(Panel A)** and uncured **(Panel B)** Hawaii flies show the same correlation of qPCR results and sequencing coverage, although the qPCR results are shifted by the amplification of genes in the bacterial genome as they are plotted against the sequencing coverage obtained from the cured insects. This indicates that the duplication of the nuwt does not occur during the tetracycline treatment of the flies to compensate for the loss of the infection. The results of the qPCR assay for coverage was compared for homozygous cured Hawaii **(Panels A)** and the offspring of a Hawaii/Mexico cross which is heterozygous for the nuwt **(Panels C)** for the 3 kbp mate pair library library. Should the Hawaii line be heterozygous, the cross should have resulted in 50% of the offspring being homozygous for absence of single copy regions of the nuwt. Instead, each of the 44 offspring (half with Hawaii mothers and Mexico fathers and half from the reciprocal cross) tested positive by PCR for single copy genes in the nuwt and the nuwt was at half of the abundance of the Hawaii line relative to internal single copy *Drosophila* nuclear gene controls in 4 of these as seen by a shifting of the y-intercept by ~1 ΔCt.

To ensure that the duplications seen in the nuwt are not an indirect effect of removing the *Wolbachia* infection or due to the tetracycline treatment itself, the relative abundance of the nuwt in whole non-tetracycline treated adult Hawaii flies was investigated (Wb+, nuwt+). These flies show the same relative levels of duplication when comparing nuwt genes, although the qPCR results are shifted 4-fold due to the additional amplification of genes in the bacterial genome (Figure 6AB). This 4-fold shifting suggests that there are four bacterial genomes for every copy of the fly chromosomes. The conservation of the relative levels of duplication in the presence of *Wolbachia* endosymbionts indicates that the extensive duplication of the nuwt has not occurred during the tetracycline treatment of the flies.

### Nuwt copy number in heterozygotes

Given that coverage can be noisy and very few regions in the nuwt were inferred to be present in a single copy, we wanted to ensure that regions with low coverage were not due to heterozygosity of the nuwt. Tet-treated flies from the Hawaii line (Wb- nuwt+) were crossed with flies from the Mexico line that does not have a *Wolbachia* infection nor any nuwt (Wb-; nuwt-). If the nuwt is heterozygous, i.e. some genes are only present on one of the chromosomes in a pair, we expect that some of the F1 offspring from the cross with a nuwt- line will lack regions identified as single copy in our coverage analysis. However, the abundance of the nuwt, including single copy regions, in the F1 offspring was half that of the parental Hawaii line using the qPCR-based coverage assay (Figure 6A,C) indicating that the nuwt is homozygous. This is not unexpected given that the Hawaii line was selected for genome sequencing because it was the most inbred line available [33].

### Nuwt copy number through the life cycle

**Figure 7:**
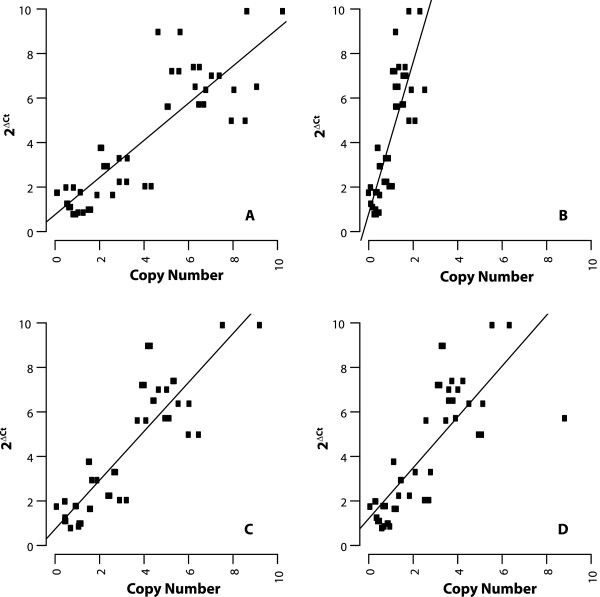
**Comparison of copy number of the nuwts through the life stages.** The copy number from the genome sequencing of adult Hawaii flies have a significant positive correlation to the qPCR results obtained for embryos **(Panel A)**, 3^rd^ instar larvae **(Panel B)**, pupae **(Panel C)**, and adults **(Panels D)**. While the slopes for embryos, pupae, and adults are approximately one, the slope for 3^rd^ instar larvae is almost four suggesting that the nuwts are 4-fold underreplicated in 3^rd^ instar larvae.

To determine whether the overrepresentation of the nuwt relative to nuclear genes could be due to something other than stable DNA duplications, we examined if there is any variation in the duplication level throughout the life cycle in the Hawaii line. Thus, the copy number of the nuwt was examined by qPCR in single individuals as embryos, third instar larvae, pupae, and adults (Figure 7). In the Hawaii line, the copy numbers were stable throughout the life cycle except in third instar larvae, where the nuwt had the same coverage pattern as the genome sequencing data, albeit with a 4-fold underrepresentation (Figure 7B) relative to single copy *Drosophila* genes. Interestingly, two *Drosophila* genes examined with the qPCR assay demonstrate the same behavior, *dsx* and an RNA polymerase III gene. In salivary glands of third instar larvae, DNA is found in the form of polytene chromosomes, which contain multiple linked chromosomes that are formed through massive replication without cell division, i.e. endoreplication [34]. During endoreplication, not all parts of the chromosome are equally replicated; instead regions like the heterochromatin become underrepresented while euchromatin can become overrepresented. Since the *dsx* gene is found in heterochromatin in *Drosophila melanogaster*[35], an underrepresentation of these genes in the larval stage might suggest they are underreplicated and hence are present in heterochromatin. While the position of *dsx* with respect to heterochromatin/euchromatin is not known in *D. ananassae*, it is consistently 2- to 8-fold underrepresented in our qPCR studies. The similar levels of underrepresentation of *dsx* and the nuwt are consistent with both being located in heterochromatin in the Hawaii line.

### Microscopy of polytene chromosomes

**Figure 8:**
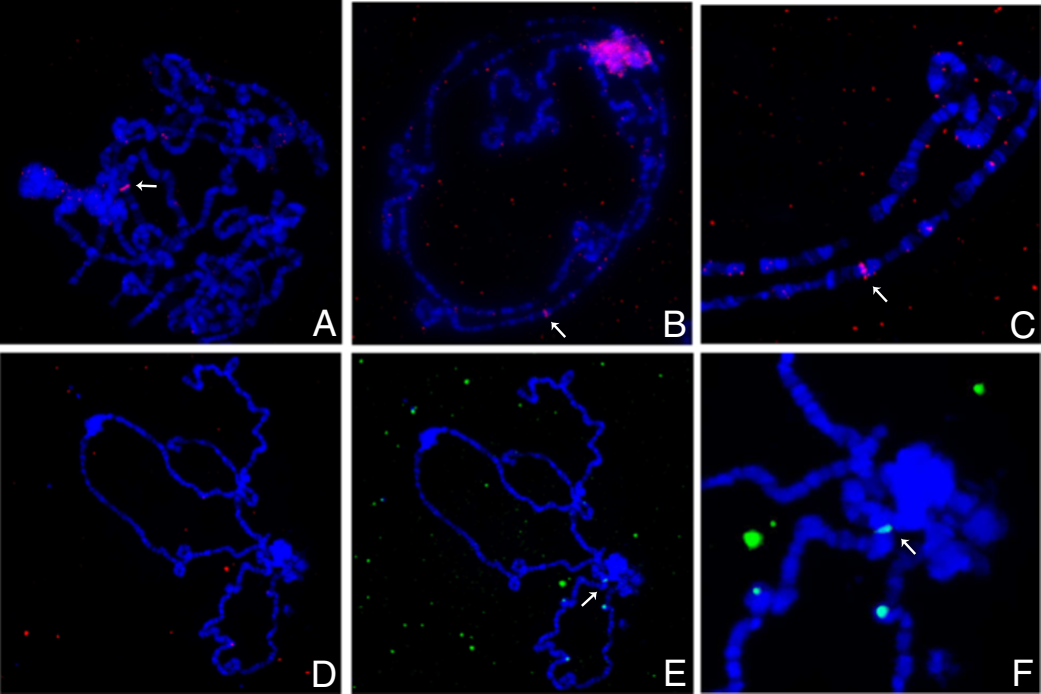
**Fluorescence in situ hybridization of polytene chromosomes.** Polytene chromosomes were hybridized with a *Wolbachia*-specific WRi_004290 probe amplified directly from genomic DNA from the Wb- Hawaii line and visualized at 40X magnification. A single band is observed and not the 8–10 bands expected **(Panel A)**. Polytene chromosomes were again hybridized with the WRi_004290 probe amplified directly from genomic DNA from the Wb- Hawaii line and visualized at 20X **(Panel B)** and 40X **(Panel C)**. A single band is observed and not the 8–10 bands expected. Polytene chromosomes were simultaneously hybridized with differentially-labelled probes for the *Wolbachia-*specific WRi_004290 (Panel **D**, pink) and a *D. ananassae actin* gene (Panels **E** &**F**, green) that were generated from sequence-verified plasmids and visualized at 40X. While hybridization by the plasmid-derived WRi_004290 probe is not detected, hybridization by the plasmid-derived actin probe is detected. This suggests that the previous detection with *Wolbachia* probes amplified directly from genomic DNA may be detecting hybridization of spurious amplification products and that the lateral gene transfer is heterochromatic.

**Figure 9:**
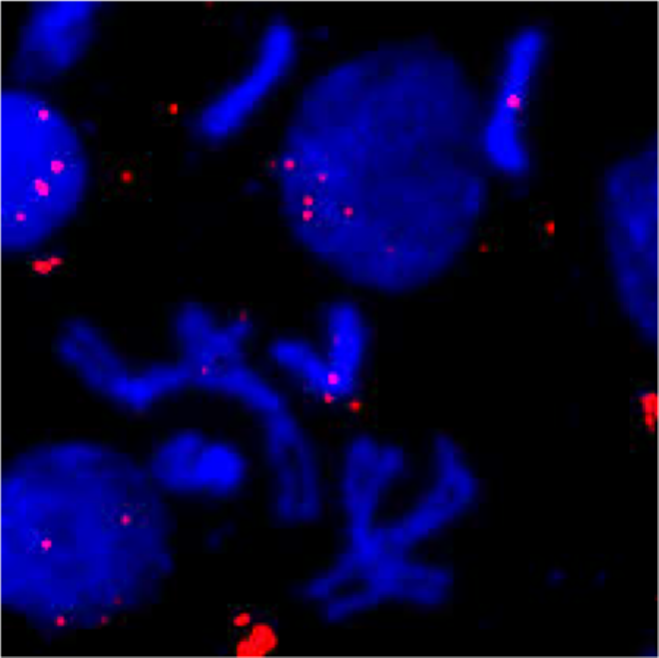
**Fluorescence in situ hybridization of mitotic chromosomes.** Mitotic chromosomes were hybridized with a *Wolbachia*-specific WRi_004290 probe amplified from sequence-verified plasmids and visualized at 60X. While hybridization was not detected in polytene chromosomes with WRi_004290, it is detected in the mitotic chromosomes. The fluorescence appears to be localized to the centromere of the abnormally large fourth chromosomes of *D. ananassae.* The larger 2^nd^ and 3^rd^ chromosomes are visualized nearby as well as a likely X chromosome. Hybridization to the fourth chromosome is consistent with the LGT being largely heterochromatic.

While qPCR on 3^rd^ instar larvae suggests that the inserts in the Hawaii line are heterochromatic, this is inconsistent with the previous results demonstrating a single band in a euchromatic region when polytene chromosomes from the salivary glands of 3^rd^ instar larvae were hybridized with a probe amplified from the nuwt for the gene homologous to WD_0484 [10]. WD_0484 is a hypothetical protein in the genome of *Wolbachia* strain *w*Mel from *D. melanogaster* with 1–2 copies per genome equivalent in the nuwt in the Hawaii line using the coverage values described above, consistent with a single band if euchromatic. If the nuwt is euchromatic, regions with 8–12 copies per genome equivalent should reveal multiple bands. Therefore, we probed polytene chromosomes from the salivary glands of 3^rd^ instar larvae from the Hawaii lines with WRi_004290 amplified from genomic DNA of the nuwt+/Wb- Hawaii line. WRi_004290 encodes the DNA repair protein RadC and is estimated to be present in 8–12 copies. Instead of seeing multiple bands in the polytene chromosomes, only a single band was revealed (Figure 8A,B,C) that appeared to be in a different location than the band for WD_0484. However, as both of these probes were prepared directly from genomic DNA, low level contaminating amplification products could result in hybridization to the wrong locus. When repeating the experiment with a probe amplified from a sequence-verified clone containing WRi_004290, no bands were identified in the polytene chromosome FISH (Figure 8D) even though a single control band was seen when using a probe with a different fluorescent label amplified directly from a sequence-verified clone containing the *D. ananassae actin* gene in the same polytene chromosome preparation (Figure 8E,F). However, when the WRi_004290 probe generated from a clone was used on mitotic chromosomes isolated from larval brain ganglia, hybridization to the heterochromatic fourth chromosome (Figure 9) was observed. Considering that the various copies of the nuwt genes co-segregate [10], we believe that all, or most, of the LGT is on the heterochromatic fourth chromosome. This is consistent with the FISH results reported here with regards to polytene chromosomes, since the fourth chromosome cannot be visualized in the polytene for *D. ananassae*[36]. Additionally, this result is more consistent with the genome sequence and mapping of scaffolds to the chromosomes in *D. ananassae*, relative to the previous results, because there is not a sufficiently large gap in the scaffold spanning the euchromatic portions of the second chromosome that could house the ~5 Mbp nuwt.

### Underrepresentation of the nuwt in the India line

The lower coverage of the nuwt in the India line relative to single copy nuclear genes indicates that it is underrepresented in the genomic DNA when compared to the majority of the *D. ananassae* genome. Unlike the putative underreplication in third instar larvae seen in the Hawaii line, underrepresentation of nuwt DNA in the India line is seen in adults. One possibility is that the population from the stock center was polymorphic for the nuwt and over time the population is losing the nuwt. Another possibility is that endoreplication is occurring or there is an episomal location for the nuwt that is underrepresented in individual flies.

A male from the untreated India line (Wb+, nuwt+) was crossed with a female from a line from Mexico that contains neither the nuwt nor any *Wolbachia* infection (Wb-, nuwt-), and an offspring was examined using the qPCR coverage assay. Such a cross should remove *Wolbachia* in a single generation without the use of antibiotics across multiple generations. The nuwt was undetectable by the F1 generation, suggesting it was lost in both the tetracycline treated and untreated populations.

Given that the India line was reared in the stock center for several years prior to us obtaining it, and that it was detected at levels like the Hawaii line at the time we obtained it, we are uncertain of what triggers this underrepresentation to occur. We re-acquired the line from the stock center and the nuwt in this line is now undetectable as well. Since the nuwt is no longer detected in the population, we could not examine this phenomenon further.

### Indonesia line, multiple copies and underrepresentation

The even coverage of the nuwt in the Indonesia line (Figure 5), as seen from the mapping of reads to the *w*Ri genome, was confirmed by the qPCR assay (Figure 2C). One concern with the even coverage observed in this line is that the sequences could stem from a residual *Wolbachia* infection, rather than from a nuclear insert. This is unlikely as the fly line was previously treated with tetracycline, and FISH on dissected ovaries using a probe for *Wolbachia* did not reveal the presence of any *Wolbachia* bacteria. However, to further confirm the presence of a nuwt in the Indonesia line, previously prepared DNA [10] from a F1 offspring of a cross between an Indonesia male and a Florida female (Wb-; nuwt-) was also examined. Such a cross should remove the maternally inherited *Wolbachia* endosymbionts. With a single insertion of the nuwt, these heterozygotes should contain half as much nuwt DNA as single copy nuclear genes. Instead, the ΔCt (average Ct for Drosophila – average Ct for nuwts) was found to be between 1.2 and 1.6 (average = 1.4; stdev = 0.18; n = 4) indicating that there is 2^1.4^×, or 2.6×, more *Wolbachia* DNA than single copy nuclear genes. Given that these are F1 hybrids, this means the parental Indonesia lines have 5.2× more *Wolbachia* DNA than single copy nuclear genes, suggesting there were originally 5 or more copies of the nuwt covering the whole *Wolbachia* genome in the Indonesia line. This may suggest that when the genome sequencing of Indonesia was undertaken it was 5-fold under-represented relative to when the F1s were generated.

While the Indonesia line with a nuwt was present in the JCDH lab when sequenced, the line has since become underrepresented for the nuwt, similar to the India line. We and others have obtained further samples of the Indonesia line from the UCSD *Drosophila* Stock Center that all now seem to lack the nuwt, as shown by our qPCR assay and other PCR-based assays (data not shown). Therefore, further experiments to explore this were not possible.

## Discussion

### Endoreplication, underreplication, and *Drosophila*origins of replication

There are multiple reasons for why not all parts of a chromosome are present in equal amounts in a cell. The most commonly known mechanism is that these different parts are not replicated an equal number of times, due to chromatin state and/or distance from origins of replication. The phenomenon of over- and underreplication is particularly common in cells that undergo endoreplication (i.e. DNA replication in the absence of mitotic division).

Apart from the endoreplication of polytene chromosomes found in salivary glands of third instar larvae, endoreplication has also been suggested to be evolutionarily conserved in ovarian follicle cells of Drosophilidae since all of the 38 species examined by Bosco *et al.* complete DNA replication with a ploidy consistent with 16 genome equivalents per cell [37]. However, in the same cells the authors also find that some DNA is represented by only 4 genome equivalents, which is consistent with the presence of heterochromatic sequences that are not replicated to the general ploidy of the cell, but instead are underreplicated [37]. While endoreplication and polytene chromosomes are best studied in third instar larva salivary glands and ovarian follicle cells in *Drosophila* species, nearly all differentiated cell types are polyploid with polytene chromosomes [38].

Given the bacterial ancestry of nuwts, it is likely that they lack *Drosophila* origins of replication. In general, if nuwts only contain a few genes, the distance from an origin of replication is inconsequential, and therefore underreplication might not occur. However, given the large size of the nuwt described here, underreplication could potentially occur merely due to a lack of *Drosophila* replication origins.

In *Drosophila*, a six-protein origin recognition complex (ORC) recognizes origins of replication, and binding of this complex is needed for replication to begin. A summary of predictions in *D. melanogaster* suggests that there are 7,329 non-overlapping ORC recognition sites with a maximum distance between ORC sites of 447 kbp in the genome that are potential origins of replication [39] (http://flypush.imgen.bcm.tmc.edu/pscreen/files/ORCsitenames.xls). Assuming that *D. ananassae* has a similar number of replication origins and that they are evenly spaced across the entire genome, one would expect to identify an origin every ~315 kbp assuming an ~231 Mbp genome, (size of the caf1 assembly [26, 33]), or every 293 kbp assuming an ~215 Mbp genome, (genome size from literature [33, 37, 40]).

Using either metric or even the maximum distance between *D. melanogaster* ORC sites, the distance between origins of replication is smaller than the size of the nuwts found in *D. ananassae.* It was previously shown that six physically distant nuwt loci co-segregate perfectly in F2 maternal inheritance crosses [10], indicating that the multiple copies of the nuwt are on the same chromosome and physically linked. Here we show that there are 2.3 *Wolbachia* reads for every 100 *Drosophila* reads in the Hawaii line (Table 1), meaning 2.3% of the *D. ananassae* Hawaii genome is from the nuwt. Assuming the *D. ananassae* genome is ~215 Mbp, this means that ~5 Mbp of the *D. ananassae* genome might consist of tandemly arrayed nuwts. Furthermore, we know of numerous integrations of retroelements [10] that make the chromosome region that is devoid of replication origins even larger, assuming origins of replication do not integrate in the nuwt along with retroelements. Therefore, the lack of *Drosophila* origins of replication may at least partially explain the underrepresentation observed in third instar larvae of the Hawaii line.

This would not explain the underrepresentation of the nuwt in the India and Indonesia lines. Although underreplication in somatic tissues, specifically the larval fat bodies and ovarian follicle cells, has been observed [38], we are not aware of any case with such high levels of underreplication as in the nuwts of the India and Indonesia lines. Therefore, while the underrepresentation of the nuwts in these two lines could result from underreplication in adult somatic tissues, we believe that they either are polymorphic in the population or that there is another, currently unknown, mechanism that explain these results. One possibility would be that nuwts are episomal or on dispensable B chromosomes in the India and Indonesia lines. However it is less clear why, before being lost, the nuwts were stable in these lines at the stock center for many years as well as in independently maintained collections.

### Origin and evolution of nuwts in the *D. ananassae genome*

Given the combination of extensive duplication containing polymorphic sites (data not shown) and the underrepresentation of the nuwt in the India and Indonesia lines yielding relatively low coverage, a robust analysis of SNPs is not feasible. However, from the coverage analysis of the nuwt in the re-sequenced lines we see a highly similar pattern of duplicated regions in the Hawaii and India lines suggesting that they share a recent common origin. Although an alternative explanation for this observation would be that the insertion of *Wolbachia* DNA in the nuclear genome is non-random, this explanation seems less likely given that the same pattern is not seen in the Indonesia line.

In the 14 lines that were originally screened in the study by Dunning Hotopp *et al.*[10] only four were found to contain the nuwt. Of these four lines, two were from South East (SE) Asia, one from India and one from Hawaii, whereas all lines from Africa, the continental Americas, and Australia as well as one additional Hawaii line lacked a nuwt. Recently, an additional 23 *D. ananassae* lines were screened, of which eight tested positive for nuwts, including three from SE Asia, one from India, one from the South Pacific island Tonga, two from Australia and one from Ethiopia (although verification of lack of bacterial infection was not performed, leaving the possibility of a residual infection) [31]. All additional nine samples from the Pacific in this screen tested negative, including one sample from Hawaii [31]. Based on both screens, it is clear that the nuwt has wide geographic distribution, but that individual lines from the same geographic regions can differ in nuwt status. Given that we have experienced loss of detection of the nuwt in the Indonesia and India lines, it is possible that some lines may have nuwts that are undetectable or that some lines might have lost the nuwt during cultivation. It is however interesting to note that all five lines from SE Asia, which likely represents the ancestral species range of *D. ananassae*[41], contain a nuwt. Colonization of other parts of the world, including India and Hawaii, could be a consequence of human activity and hence these populations could represent independent migrations from the same ancestral population or human-enabled migrations between the two locations. Hence, integration of *Wolbachia* DNA into the nuclear genome of *D. ananassae* might have occurred just once and then spread to different locations via human activity. One possible scenario is thus that the most recent common ancestor of all three lines (Hawaii, India and Indonesia) was a line that had extensive duplication of the whole *Wolbachia* genome and that the Indonesia lineage maintained the complete copies of these integrated genomes while the lineage leading to Hawaii and India lost some parts of the nuwt.

The chromosomal location of the integration site of all three nuwts could inform our understanding of how nuwts form and perhaps also about their ancestry. However, due to the integration of numerous host retrotransposons inside the nuwt, we could not identify its exact location based on sequence data in any of the lines despite numerous attempts. Although FISH on mitotic chromosomes could be used to locate the nuwt in the Hawaii line to chromosome four, the inability to detect the nuwt in the two other lines currently eliminates the possibility of exploring this further.

### Chromosome four

In most *Drosophila* species, chromosome four (Muller element F) is referred to as the *dot* chromosome because of its small size. In *D. melanogaster,* chromosome four is the size of a typical bacterial genome at ~4.2 Mbp, containing 92 genes and ~3.0 Mbp of repeated sequences [42]. Most *Drosophila* species sequenced have a similarly sized fourth chromosome with only 1.0-3.4 Mbp of contigs mapped to it [33, 42]. Interestingly, even though part of chromosome four can normally polytenize in most *Drosophila* species, the whole chromosome shows properties similar to heterochromatin.

In contrast, the fourth chromosome in *D. ananassae* is equivalent in size to the 37 Mbp X chromosome (Muller element A) [33] and has not been observed to polytenize. The increased size of the fourth chromosome of *D. ananassae* is thought to be the result of an expansion of repeats, with 32.5% of the sequence arising from retro-elements (27.1%) and other DNA-based repeats (5.4%) [33]. However, the real repeat content is probably even higher, since gaps are likely to contain even more repeats. Based on our results, we propose that the ~5 Mbp of nuwt sequence described here, as well as the retro-elements known to be integrated into the nuwt, are located on the fourth chromosome of *D. ananassae*. The nuwt would thus represent ~20% of the genetic material in chromosome four and partially explain its larger size in *D. ananassae*. However, as there is no described size variation of chromosome four in *D. ananassae*, although there is variation in the presence of the nuwt, it might just indicate that this species is prone to accumulate genetic material on this chromosome and perhaps also in general, since *D. ananassae* has the highest repeat content of the sequenced *Drosophila* species [43].

## Conclusions

In conclusion, we find that multiple *Wolbachia* genomes are transferred to the *Drosophila ananassae* nuclear genome. The Hawaii and India lines show uneven coverage suggesting that different parts of these nuwts are duplicated to different levels whereas the even coverage seen across the nuwt in the Indonesia line suggests nearly perfect duplicates. Furthermore, the large nuwt in the Hawaii line becomes underreplicated in third instar larvae suggesting that it is located in heterochromatin. However, the underrepresentation of the nuwt in the India and Indonesia lines is more severe than for heterchromatic regions and might reflect a gradual loss of the nuwt in the lab population or a yet unknown mechanism causing underrepresentation of DNA. Hybridization to mitotic chromosomes indicates that the nuwt is located on the fourth chromosome, which is enlarged in *D. ananassae* compared to other *Drosophila* species. Taken together with low levels of expression from genes in the nuwt [10, 25], it seems unlikely that the nuwt genes are functional. This might explain why the nuwt has not spread to more populations and other continents. However, it is still possible that the nuwts are functional under a yet unknown condition, which could explain their presence in multiple geographic locations. Taken together, each line is unique in its combination of attributes and present interesting aspects for further research on LGT between symbionts and their hosts.

## Methods

### *Drosophila ananassae*lines

The Wb + nuwt + Hawaii (14024–0371.13), India (14024–0371.31), Indonesia (14024–0371.34) lines and Wb- nuwt- Mexico (14024–0371.00) and Florida (14024–0371.12) lines were obtained from the *Drosophila* Species Stock Center (University of California, San Diego, San Diego, CA, USA). Populations were grown on Jazz-Mix *Drosophila* food (Applied Scientific, Waltham, MA, USA) in plastic bottles at room temperature and humidity, or in an insect growth chamber (Caron, Marietta, OH, USA) at 25°C and 68% humidity.

### Tetracycline curing

All Wb + nuwt + lines were treated with tetracycline to remove the *Wolbachia* endosymbiont and generate Wb- nuwt + versions. Populations were grown for at least two generations on Jazz-Mix media containing 0.025% (25 mg per 100 mL media) tetracycline (Teknova, Hollister, CA, USA). Absence of the *Wolbachia* endosymbiont in the lines was confirmed by FISH microscopy on ovaries using *Wolbachia*-infected insects as a positive control.

### Fluorescence *in situ*hybridization for assessing *Wolbachia*colonization status

Flies were examined by FISH to verify presence or absence of *Wolbachia* endosymbionts. Freshly dissected ovaries were fixed in methanol for 1 h at 4°C and washed twice in 1× PBS. To confirm *Wolbachia* infection, two Alexa Fluor 488-labeled *Wolbachia*-specific oligonucleotides (W1: AACCCGGCCGAACCGACCC; W2: CTTCTGTGAGTACCGTCATTATC) were hybridized to the fixed ovaries at 37 °C overnight in hybridization buffer containing 50% formamide, 5× SSC, 200 g/L dextran sulfate, 0.5× Denhardt’s solution, 0.1 M dithiothreitol (DTT), 250 mg/L salmon sperm DNA, 250 mg/L tRNA, 250 mg/L poly(A). Hybridized ovaries were washed in 1× SSC + 10 mM DTT at room temperature once followed by 1× SSC + 10 mM DTT at 55 °C twice, in 0.5× SSC + 10 mM DTT at 55°C twice, and in 0.5× SSC + 10 mM DTT at room temperature. Ovaries were placed on a microscope slide and mounted with glycerol, a drop of SlowFade Gold antifade reagent (Invitrogen, Grand Island, NY, USA), stained with DAPI, and visualized with a Nikon TE-2000 using inverted fluorescence microscopy (Nikon, Melville, NY, USA).

### Genome sequencing

For the India x Florida cross (I × F) sequenced at IGS, DNA was isolated from ~100 larvae in 6 different extractions using the Gentra Puregene Tissue kit (Qiagen, Valencia, CA, USA) with an incubation with Proteinase K (Finnzymes, Vantaa, Finaland) for at least 4 h, but in several cases much longer. All samples were re-purified so that protein precipitation was done twice. For the DNA sequencing conducted by NCGR, DNA was extracted from 20 larvae with the Aqua pure tissue kit (Bio-Rad, Solna, Sweden) with a proteinase K incubation for 3.5 h at 55°C followed by an overnight incubation at room temperature. In addition, the protein precipitation was repeated for this sample. For all other samples, DNA was isolated using with a DNeasy Blood & Tissue Kit (Qiagen, Germantown, MD, USA). Up to 50 mg insects were ground in liquid nitrogen with a disposable mortar and pestle and then processed according to the standard DNeasy protocol.

The library sequenced at NCGR was prepared using standard Illumina protocol for mate pair library preparation. For all other DNA samples, both a 300-bp paired-end and a ~3000-bp mate-pair library were constructed for sequencing on the Illumina platform using the NEBNext® DNA Sample Prep Master Mix Set 1 (New England Biolabs, Ipswich, MA, USA). First, DNA was fragmented with the Covaris E210. Then libraries were prepared using a modified version of manufacturer’s protocol. The DNA was purified between enzymatic reactions and the size selection of the library was performed with AMPure XT beads (Beckman Coulter Genomics, Danvers, MA). The PCR amplification step was performed with primers containing a 6 bp index sequence. Sequencing was performed using both an Illumina Genome Analyzer IIx and an Illumina HiSeq2000. Because the experiments were conducted during a period of rapid evolution of the Illumina sequencing technology and the read-length was maximized at each stage, the paired-end data here represents reads that range from 57 bp to 101 bp (Table 1). Conversely, in order to minimize the number of chimeric sequences generated by sequencing across the untagged junction in the mate-pair libraries, the reads for these libraries were limited to between 35–40 bp (Table 1). Base calling and quality scoring was performed using Illumina software followed by in-house quality assessment and control pipelines to truncate and eliminate poor-quality reads. Sequencing data is available in the SRA (Hawaii line: SRA052201; Indonesia: SRA052189; India: SRA052191; I × F: SRA052193).

### Sequence analysis

Reads were mapped against the *D. ananassae* caf1 assembly [26] and the *w*Ri *Wolbachia* genome [27] using BWA [28] version 0.5.9-r16 with the default parameters except that the maximum number of alignments reported in the XA tag was 1000. Reads from the mate pair libraries were reverse complemented prior to alignment using fastx_reverse_complement (http://hannonlab.cshl.edu/fastx_toolkit/). Duplicate sequences were removed and reads were position sorted using Picard version 1.48 (http://broadinstitute.github.io/picard/) with the default parameters. Genome sequencing coverage was calculated with MPILEUP in SAMTOOLS version 0.1.19-44428 cd [44] using the default parameters. Copy number was calculated by dividing the coverage by the mode of the coverage using a bin size of 1. Plots were generated using the R statistics and analysis package version 2.15.2.

Given the shared ancestry of mitochondria and *Wolbachia,* we wanted to ensure that reads were not mapping to both. Across all three datasets, only nine reads were identified that mapped to both locations (1 in Hawaii, 0 in India, and 8 in Indonesia). Given these very low numbers that would not influence the values presented or the interpretation, we did not consider cross mapping to be an issue to address further. The only regions where we saw erroneous mapping of reads were in the rRNA genes as discussed in the text, which likely arose because fed adults with an active gut microbiome were used for preparing genomic DNA.

### Quantitative PCR (qPCR)

Relative coverage of the *Wolbachia* insert in the *Drosophila* genome was examined with qPCR on genomic DNA. Primers (Additional file 1) were designed based on sequence coverage and loci were selected that were single copy in the *w*Ri genome. Primers were designed using Primer3 and synthesized by Sigma-Aldrich (St. Louis, MO, USA). Dilutions of genomic DNA (0.2-1 μL stock gDNA per 20 μL reaction) were used as templates in a qPCR reaction containing 2× QuantiTect SYBR Green (Qiagen, Germantown, MD, USA), RNase-free water, and coverage primers using the standard protocol. The assays were conducted using an ABI 7900 instrument (Applied Biosystems, Foster City, CA, USA). The reactions were denatured at 95°C for 15 min followed by amplification with 45 cycles of 94°C for 15 s, 55°C for 30 s and 72°C for 30 s. Reactions were followed by a melt curve analysis that starts at 55°C, with a dissociation step at 95°C for 1 min plus 0.5°C/cycle for 80 cycles. Comparisons of normalization by (a) aggregating the results for the six single copy *Drosophila* genes or (b) using each individual single copy nuclear *Drosophila* gene showed little difference since the six single copy *Drosophila* genes routinely have <1 Ct variance. Linear regression analysis was completed using the standard R statistical package.

### Fluorescent probe synthesis from genomic DNA

Genomic DNA was isolated from tetracycline-treated *D. ananassae* Hawaii flies using the Qiagen DNeasy Kit (Qiagen, Valencia, CA, USA). The PCR DIG Probe Synthesis Kit (Roche Applied Science, Indianapolis, IN, USA) was used to synthesize probes from 25 ng of genomic DNA with primers specific for a highly duplicated region of the nuwt (WRi_004290), as indicated by whole genome sequencing and qPCR. The labeled probe was purified using the Qiagen Qiaquick PCR Purification Kit and suspended in 150 μL hybridization buffer consisting of 2× saline-sodium citrate (SSC), 10% dextran sulfate, 50% formamide, and 0.8 mg/mL salmon sperm DNA.

### Fluorescent probe synthesis from plasmid DNA

Genomic DNA was isolated from tetracycline-treated *D. ananassae* Hawaii flies using the Qiagen DNeasy Kit (Qiagen, Valencia, CA, USA) and used as a template for PCR with NEB 2× Master Mix (New England Biolabs, Ipswich, MA), using primers specific for a highly duplicated region of the nuwt (WRi_004290), as indicated by whole genome sequencing and qPCR. The amplicon was cloned into a TOPO TA subcloning vector (Life Technologies, Grand Island, NY, USA). Plasmid DNA was isolated with the Qiagen Qiaprep Miniprep Kit. For the digoxigenin probes, 50 pg was used as a template in a reaction using the PCR DIG Probe Synthesis Kit (Roche Applied Science, Indianapolis, IN, USA) with primers specific for WRi_004290. The labeled probe was purified using the Qiagen Qiaquick PCR Purification Kit and suspended in 150 μL hybridization buffer consisting of 2× saline-sodium citrate (SSC), 10% dextran sulfate, 50% formamide, and 0.8 mg/mL salmon sperm DNA. For the biotinylated probes, 1 μg of purified plasmid was used as a template in the Roche biotin high prime labeling protocol, following the manufacturer’s instructions, and suspended in 150 μL hybridization buffer as described above.

### Polytene chromosome preparation

Salivary glands were dissected in 1× phosphate buffered saline (PBS) and incubated in 40 μL 45% acetic acid for 2 min followed by incubation in 10 μL 1:2:3 lactic acid:water:glacial acetic acid for 3 min on an 18 mm × 18 mm cover slip. The glands and cover slip were picked up with a SuperChip poly-L-lysine slide (Thermo Scientific, Waltham, MA, USA), flipped over, and the areas of the glands were gently tapped with forceps, followed by tapping concentric circles in an outward spiral. Excess fixative was blotted from an inverted slide applying gentle pressure. The face-up slide was gently streaked rapidly in a zig-zag manner in one plane, rotated 90°, and rapidly streaked again. Excess fixative was again blotted off, and with the cover slip facing down, the slide was squashed firmly with a stack of cleaning tissues. The spreads were checked with a microscope for refractivity. The slides were incubated at 4°C overnight, immersed in liquid nitrogen for 15 s, and the cover slip was flipped off using the edge of a razor blade as a lever. Slides were then incubated in 96% ethanol for 10 min, air dried, and checked for refractivity.

### Mitotic chromosome preparation

Brains were dissected from the 3^rd^ instar of *D. ananassae* Hawaii larvae in 1% sodium citrate and incubated for 10 min at room temperature. The ganglia were then incubated in 3:1 methanol:glacial acetic acid for 30 s, 45% acetic acid for 30 s, and 60% acetic acid for 4 min on a poly-L-lysine slide under an 18 mm × 18 mm cover slip. The slide was then placed between tissue paper and the perimeter was tapped with an eraser. Excess fixative was again blotted off, and with the cover slip facing down, the slide was squashed firmly with a stack of cleaning tissues. The spreads were checked with a microscope for refractivity. Slides were immersed in liquid nitrogen for 15 s, and the cover slip was flipped off with a razor blade. Slides were then incubated in cold 70% ethanol for 5 min, cold 80% ethanol for 5 min, and cold 100% ethanol for 10 min, air dried, and checked for refractivity.

### Probe hybridization for fluorescence *in situ*hybridization

Chromosome preparations were incubated in 2× SSC for 1 h at 70°C and dehydrated in 70% ethanol for 5 min, 96% ethanol for 5 min, and air dried. The preparations were denatured in 0.07 NaOH for 10 min and washed in 2× SSC for 1 min, 1 min, and 5 min. The slides were then dehydrated as described above. The DIG- and/or biotin-labeled probes were incubated at 95°C for 5 min, snap cooled on ice, and warmed to 37°C. A volume of 10 μL probe was loaded onto the chromosome preparation under an 18 × 18 mm coverslip and incubated at 37°C for 16–20 h. [45]. Following the overnight incubation, slides were washed three times in 2× SSC for 5 min at 37°C followed by one wash in 1× PBS for 10 min. Secondary, or indirect, labelling for single fluorophore and double fluorophore hybridizations were conducted as described separately below. Subsequently, preparations were stained with 4′,6-diamidine-2′-phenylindole dihydrochloride (DAPI) (Roche Applied Sciences, Indianapolis, IN, USA) for 10 min and washed briefly in 1× PBS, mounted with SlowFade Gold Antifade Reagent (Life Technologies, Grand Island, NY, USA) under a cover slip, and imaged with a Nikon TE2000 fluorescence microscope (Nikon Instruments, Melville, NY, USA).

### Single fluorophore FISH

For detection of DIG-labeled probes, chromosomes were incubated with 1:250 mouse anti-DIG primary antibody (Roche Applied Science, Indianapolis, IN, USA) in 1% BSA and 2 mg/mL RNase A in 1× PBS for 1 h, followed by washes in 1× PBS for 5 min, 1× PBS + 0.1% Triton X-100 for 5 min, and 1× PBS for 5 min. Chromosomes were then incubated with 1:500 Alexa 488 goat anti-mouse secondary antibody (Life Technologies, Grand Island, NY, USA) in 1% BSA and 2 mg/mL RNase A in 1× PBS for 1 h, followed by washes in 1× PBS for 5 min, 1× PBS + 0.1% Triton X-100 for 5 min, and 1× PBS for 5 min.

### Dual fluorophore FISH

For simultaneous detection of DIG- and biotin-labeled probes, 1:250 Alexa 555-streptavidin primary antibody (Life Technologies, Grand Island, NY, USA) was combined with the mouse anti-DIG antibody in 1% BSA and 2 mg/mL RNase A in 1× PBS for 1 h, followed by washes in 1× PBS for 5 min, 1× PBS + 0.1% Triton X-100 for 5 min, and 1× PBS for 5 min. Chromosomes were then incubated with 1:500 Alexa 488 donkey anti-mouse secondary antibody (Life Technologies, Grand Island, NY, USA) and 1:250 goat biotinylated anti-streptavidin secondary antibody (Vector Laboratories, Burlingame, CA, USA) in 1% BSA and 2 mg/mL RNase A in 1× PBS for 1 h, followed by washes in 1× PBS for 5 min, 1× PBS + 0.1% Triton X-100 for 5 min, and 1× PBS for 5 min. The signal was again amplified with 1:500 Alexa 488 donkey anti-mouse secondary antibody and 1:500 Alexa 555-streptavidin primary antibody in 1% BSA and 2 mg/mL RNase A in 1× PBS for 1 h, followed by washes in 1× PBS for 5 min, 1× PBS + 0.1% Triton X-100 for 5 min, and 1× PBS for 5 min.

### Availability of supporting data

The data sets supporting the results of this article are available in the Sequence Read Archive repository: SRP012548, SRP012550, SRP012551, and SRP012555.

## Electronic supplementary material

Additional file 1: **Primer sequences for qPCR experiments.** Excel spreadsheet of primer sequences used in qPCR experiments. (DOCX 17 KB)

## Abbreviations

DTT: Dithiothreitol
FISH: Fluorescence *in situ* hybridization
I × F: India x Florida
LGT: Lateral gene transfer
numt: Nuclear mitochondrial transfers
nuwt: Nuclear *Wolbachia* transfers
ORC: Origin recognition complex
qPCR: Quantitative PCR
SE: South East
SSC: Saline-sodium citate
tet: Tetracycline
Wb: *Wolbachia*.
