# Supplementary material for: Extensive duplication of the Wolbachia DNA in chromosome four of Drosophila ananassae
Source: BMC Genomics. 2014 Dec 12;15(1):1097. doi: 10.1186/1471-2164-15-1097 (PMC4299567; doi:10.1186/1471-2164-15-1097)
Supplement: Supplementary file 1 — Additional file 1: Primer sequences for qPCR experiments. Excel spreadsheet of primer sequences used in qPCR experiments. (DOCX 17 KB) [file 12864_2014_6901_MOESM1_ESM.docx]

**Additional file 1. Primers for Quantitative PCR.**

| **Name** | **F-Primer** | **R-Primer** | **Product Size** |
| --- | --- | --- | --- |
| 5×_UNIQ_1 | TGCAACAAGATATCCCCATTAAC | TCACATCCCATTTCCTACCG | 159 |
| 5×_UNIQ_2 | GCAATGAAACGAGGTTCAGC | GCTGGACCTTTGAATGGAAC | 104 |
| 5×_UNIQ_3 | GGAATGTCAATTGTTGTGATGG | TGCATTTTGAAATTCTATAGTGTTAAG | 198 |
| 5×_UNIQ_4 | TCTGGGGTTTTCATTGCTTC | GCCAGCGAGGAATTCAATAG | 131 |
| 5×_UNIQ_5 | TTTATGCAGCCTAGCGTTACC | TGCGTTAGATTGCGCTATTG | 112 |
| 10×_UNIQ_1 | CACTTCACCTTTTCGGAAGC | TTTGAGCTTTCGGTATCACG | 147 |
| 10×_UNIQ_2 | GACAGTGAAGATTATTGGTTTGGTC | TGCAATCATAAAAGCAATTAACG | 186 |
| 10×_UNIQ_3 | ACCGCAAAGTGTGAGGTAAC | TGTGCCTGTAGTCATTGGTG | 136 |
| 10×_UNIQ_4 | TTTCCTGTCGTTTTATGTGAGAAG | TTAAAGCGGCAGTTGAAATC | 100 |
| 10×_UNIQ_5 | TAACCGAAACACCTGCAACC | AAAGGCGTACGCACTGGTAG | 106 |
| 20×_UNIQ_1 | ACTTGAATTGTGGGCAAACC | ACCTGCATTAAAGGCCTGAG | 144 |
| 20×_UNIQ_2 | CCATGTGGAAACACCAACTG | AATCACGATTCCCATGATCC | 105 |
| 20×_UNIQ_3 | TTCGGCAGGTCAATCTTAGG | GTTCAACATTTCCTGCAACG | 127 |
| 20×_UNIQ_4 | CATTTCCTTCCACTCCAAGC | AGCGAAAGATGGAAGTGGTG | 139 |
| 20×_UNIQ_5 | TGCCAAGCGAAAAGACTATTG | TTTTCATCTTCTGTTATCGTTCG | 131 |
| 40×_UNIQ_1 | AAGTCAACAATGCCCTCCTG | TGCTATTGGCGTCTCAAGTG | 186 |
| 40×_UNIQ_2 | TTTTCGGCAGTTTTAGTGAGG | CCCCCTATCGCGTTCTTATC | 113 |
| 40×_UNIQ_3 | TTGGAACTTGCAAATGATGG | GCCCCTTTATCAAGTTGAGC | 122 |
| 40×_UNIQ_4 | CTTGCAGCAGAGTCAACAGC | TCAACAGGGTTTCGTACAGG | 127 |
| 40×_UNIQ_5 | ATTGGCACAGTTCCTTTTGC | GGTATTAGGAATAGGGGTGGTG | 107 |
| 60×_UNIQ_1 | TCGAAGTGGTCATTTGATATACG | TCCTCATCCTCCTTTGATGG | 171 |
| 60×_UNIQ_2 | CCAGGTTTTGCATTCTTTCC | GCTGCCTGATTTAACCGAAC | 162 |
| 60×_UNIQ_3 | TATGTCCACATTGCCGTCAG | ACTCTTTGGTGATGGTGCTG | 100 |
| 60×_UNIQ_4 | CTGCTTCTGAAAGAGCAAAGG | TTTCGCGATGAAAGTTGTTG | 165 |
| Dana_UNIQ_1 | CTGAGCTGCGAATACTGCAC | CAAGTCCGGCTTAATCTTGG | 186 |
| Dana_UNIQ_2 | TCTCCAGCTAAAAGCGAAGC | TCCTGTGGAGTGTTTTGCTG | 158 |
| Dana_UNIQ_3 | GGTTATCTCCACGGGTATGC | TTCGGGCATAGTTGGGTAAG | 124 |
| Dana_UNIQ_4 | ACATTACCACGGAGGTGGAG | TTCTTGACGAGATGCCTGTG | 172 |
| Dana_UNIQ_5 | CTTGGCGGGAGTACGTAAAG | ATGCCAGTGCGATAGAATCC | 178 |
| Dana_UNIQ_6 | ATCTGGCGGTGTTCTGTTTC | CTGCATGACTTCGCACAAAC | 158 |
| Dana_UNIQ_7 | GTTCTCCTGGTGGCACATTG | GTCACCGGTCGATACTCCAG | 150 |
